# Viral Metagenomic Analysis Reveals a Human Rhinovirus from Hospitalized Neonates

**DOI:** 10.1128/MRA.00106-21

**Published:** 2021-04-15

**Authors:** Hong Li, Yuqing Xiao, Jinquan Lü, Wen Zhang, Hongyan Lu

**Affiliations:** a Department of Pediatrics, Affiliated Hospital of Jiangsu University, Zhenjiang, Jiangsu, China; b School of Medicine, Jiangsu University, Zhenjiang, Jiangsu, China; KU Leuven

## Abstract

Here, the coding-complete genome of a human rhinovirus (HRV) belonging to the HRV-A clade was determined from a pool containing nine nasopharyngeal secretion specimens from hospitalized neonates. PCR screening indicated that this HRV variant was present in a cohort of 45 hospitalized neonates, with a positivity rate of 11.1% (5/45 patients).

## ANNOUNCEMENT

Human rhinoviruses (HRVs) are members of the genus *Enterovirus*, belonging to the family *Picornaviridae*, and are small, positive-sense, nonenveloped RNA viruses. HRVs are responsible for the majority of acute wheezing illnesses in infants and children (1–3). Three HRV species (A, B, and C) are recognized by the International Committee on Taxonomy of Viruses (ICTV), and they are further subdivided into approximately 170 genotypes (4).

In this study, in order to investigate the viruses in the upper respiratory tract of hospitalized neonates using viral metagenomics, 45 nasopharyngeal secretion samples were collected from 45 hospitalized neonates who were admitted to the Neonate Department of the Affiliated Hospital of Jiangsu University (Zhenjiang, China) in September to December 2018. The samples were collected with informed written consent from the parents of each neonate. The 45 samples were randomly pooled into 5 sample pools, with each pool including 9 samples. Each nasopharyngeal secretion sample was individually resuspended in 10 volumes of phosphate-buffered saline (PBS) and vigorously vortex mixed for 5 min. After centrifugation (10 min at 15,000 × *g*), the supernatant was collected and stored at −80°C until nucleic acid extraction. The supernatant (450 μl) of each pool (50 μl per sample) was filtered through a 0.45-μm filter (Millipore) to remove eukaryotic and bacterial cell-sized particles, and 200 μl of the filtrates was then treated at 37°C for 60 min with a mixture of DNase and RNase (Life Technologies) to digest unprotected nucleic acids (5). Viral nucleic acids in the filtrates were then extracted using the QIAamp MinElute virus spin kit (Qiagen) according to the manufacturer's protocol. Five libraries were constructed using the Nextera XT DNA sample preparation kit (Illumina) and sequenced using the Illumina MiSeq platform with 250-bp paired-end reads with dual barcoding for each pool. For bioinformatic analysis, paired-end reads of 250 bp generated by the MiSeq system were debarcoded using software from Illumina (6–8). The total numbers of sequencing reads for the five sample pools were 106,004, 50,226, 66,438, 53,482, and 30,916. Clonal reads were removed, and bases with a low Phred score (below 10) were trimmed. Adaptors were trimmed using the default parameters of VecScreen, which uses National Center for Biotechnology Information (NCBI) BLASTn with specialized parameters designed for adapter removal. The cleaned reads were *de novo* assembled by SOAPdenovo2 v.r240 using a kmer size of 63 with default settings. The assembled contigs, along with singlets, were compared with an in-house viral proteome database using BLASTx with an E value cutoff of <10^−5^ (9).

**FIG 1:**
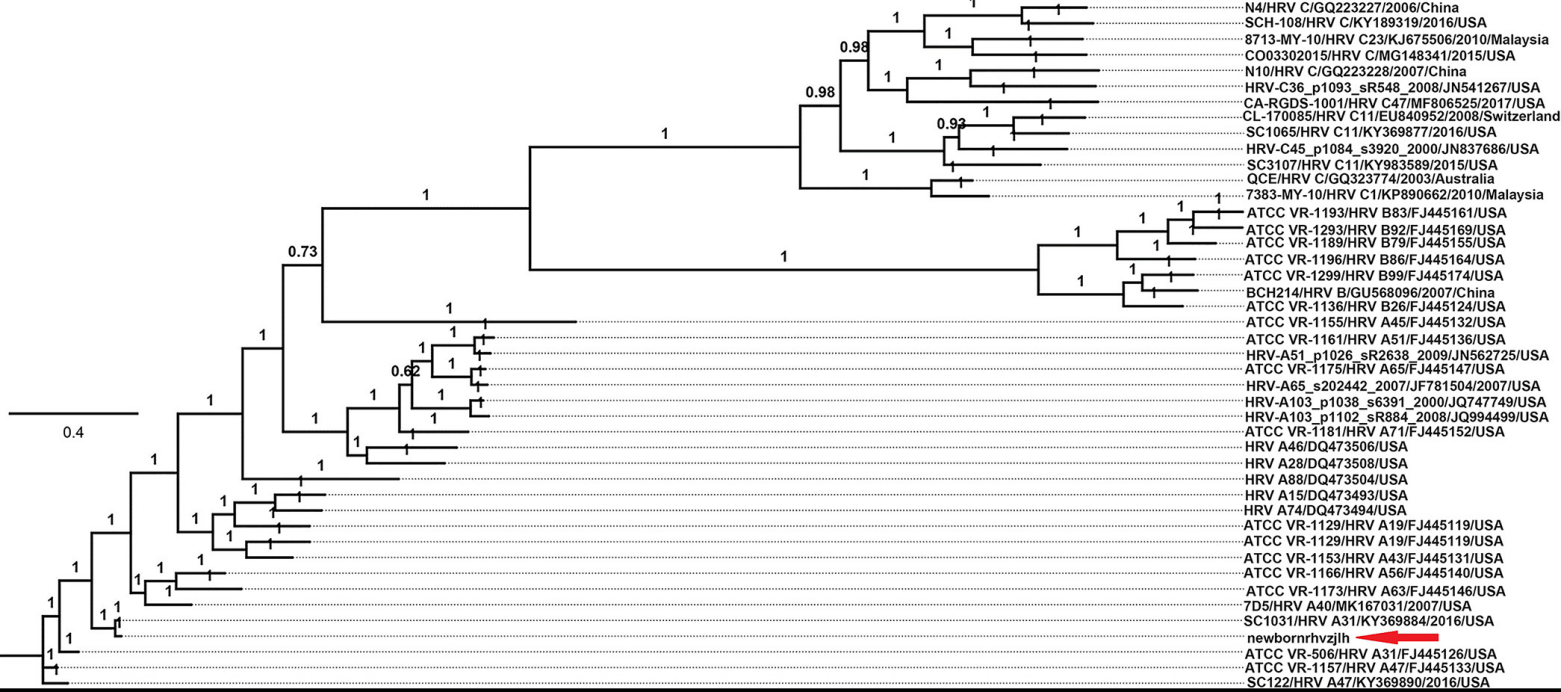
Phylogeny of the HRV genome detected in nasopharyngeal secretion specimens from hospitalized neonates. Phylogenetic analysis was based on the coding-complete genome of the HRV identified here and other representative HRV strains. The phylogenetic tree was constructed by using MrBayes v3.2. The Markov chain was run for a maximum of 1 million generations; every 50 generations were sampled, and the first 25% of Markov chain Monte Carlo (MCMC) samples were discarded as burn-in, with a stopping rule implemented so that the analysis would halt when the average deviation of the split frequencies was less than 0.01%. The HRV identified here is marked with a red arrow.

Results indicated that sequences showing similarity to HRVs were found in one library (identification no. newbornoral55 [SRA accession no. SRX7070536]), which contained two large contigs with sequence lengths of 4,635 bp and 1,569 bp; BLASTn searching in GenBank indicated that the two contigs showed similarity to the 5′ and 3′ regions of HRV A31, respectively, suggesting that the two contigs were from the same genome of a single HRV. PCR primers bridging the gap between the two contigs were then designed. Reverse transcription-PCR (RT-PCR), followed by Sanger sequencing, was performed. The Sanger reads and the prior two contigs were assembled using the default parameters in Geneious v.11.1.2 to obtain a coding-complete genome. The coding-complete genome of this HRV (named newborn_rhvzjlh) was determined from one sample and had a length of 7,033 bp, including 5′-terminal noncoding sequences (538 bp) and 3′-terminal noncoding sequences (22 bp), with a GC content of 38.2%. A BLASTn search showed that this HRV belongs to HRV-A, sharing 97.0% sequence identity with an HRV A31 strain (GenBank accession no. KY369884). Using appropriate coding-complete reference genomes from GenBank, an alignment was performed using MUSCLE in MEGA v.7.0 (10); subsequently, a phylogenetic tree was generated using MrBayes v.3.2 (11, 12), confirming the clustering of strain newborn_rhvzjlh in the group HRV-A (Fig. 1).

To investigate the prevalence of HRV-A in children, viral nucleic acid was extracted from all of the 45 individual samples using the TaKaRa MiniBEST universal RNA extraction kit. A set of primers targeting the VP4-VP2 gene region of this HRV was designed and used for nested PCR screening of the whole set of 45 nasopharyngeal secretion samples. Primers CCGGCCCCTGAATGYGGCTAA and ACATRTTYTSNCCAAANAYDCCCAT were used for the first round of PCR, and primers ACCRACTACTTTGGGTGTCCGTG and TCWGGHARYTTCCAMCACCANC were used for the second round; the expected length of the amplified fragment was 563 bp. Results indicated that 11.1% of the samples (5/45 samples) were positive. The PCR amplicons were sequenced with the Sanger method.

In brief, we found an HRV-A strain among children in Zhenjiang, China, and characterized its coding-complete genome. Phylogenetic analysis indicated that this HRV clustered with an HRV A31 strain (GenBank accession no. KY369884). Our epidemiological data indicated that this HRV-A variant was prevalent among children in this area.

### Data availability.

The coding-complete genome of the recombinant HRV determined here was deposited in GenBank with the accession no. MT157224.1. The raw sequencing reads generated by next-generation sequencing (NGS) from nasopharyngeal secretion samples from neonates were deposited in the GenBank Sequence Read Archive (SRA), and the accession numbers are SRX7070391, SRX7070404, SRX7070494, SRX7070535, and SRX7070536.
